# The Role of *RNF213* 4810G>A and 4950G>A Variants in Patients with Moyamoya Disease in Korea

**DOI:** 10.3390/ijms18112477

**Published:** 2017-11-21

**Authors:** Young Seok Park, Hui Jeong An, Jung Oh Kim, Won Seop Kim, In Bo Han, Ok Joon Kim, Nam Keun Kim, Dong-Seok Kim

**Affiliations:** 1 Department of Neurosurgery, College of Medicine, Chungbuk National University, Cheongju 28644, Korea; youngseokparkmd@gmail.com; 2 Department of Biomedical Science, College of Life Science, CHA University, Seongnam 13488, Korea; tody2209@naver.com (H.J.A.); jokim8505@gmail.com (J.O.K.); 3 Department of Pediatrics, College of Medicine, Chungbuk National University, Cheongju 28644, Korea; wskim@chungbuk.ac.kr; 4 Department of Neurology, CHA Bundang Medical Center, School of Medicine, CHA University, Seongnam 13496, Korea; haninbo@gmail.com (I.B.H.); okjun77@cha.ac.kr (O.J.K.); 5 Department of Pediatric Neurosurgery, Severance Hospital, Seoul 03722, Korea; dskim@yuhs.ac

**Keywords:** moyamoya disease, single nucleotide polymorphism, genetic, stroke, cerebrovascular disease, *RNF213*

## Abstract

Although a founder variant of *RNF213* 4810G>A is a major genetic risk factor for moyamoya disease (MMD) in East Asians, the frequency and disease susceptibility of *RNF213* variants remain largely unknown. This study investigated the mutation analysis of *RNF213* (4448, 4810, 4863, and 4950) between Korean MMD and healthy controls. We performed a polymerase chain reaction-restriction fragment length polymorphism analysis. To identify the association between *RNF213* gene polymorphisms and MMD disease, we performed statistical analyses such as multivariable logistic regression and Fisher’s exact test. Genetic data from 117 MMD patients were analyzed and compared with 253 healthy controls. We assessed and compared single nucleotide polymorphisms of *RNF213* (4448, 4810, 4863, and 4950) between MMD and control groups. We performed genome-wide association studies to investigate the genetic pathophysiology of MMD. Among the *RNF213* variants (4448G>A, 4810G>A, 4863G>A, and 4950G>A), *RNF213* 4810G>A and 4950G>A variants were more frequent in MMD patients. In a subgroup analysis, the *RNF213* 4810G>A was more frequent in moyamoya disease, and the comparison with GG+AA genotype was also significantly different in moyamoya patients. These results confirm that *RNF213* 4810G>A and *RNF213* 4950G>A were more frequent in MMD patients. We have confirmed that *RNF213* 4810G>A and 4950G>A are strongly associated with Korean MMD in children and adults as well as for the ischemic and hemorrhagic types.

## 1. Introduction

Moyamoya disease (MMD) is a chronic cerebrovascular occlusive disorder that results in transient ischemia, cerebral infarcts, and hemorrhagic strokes [1,2,3,4,5]. MMD occurs worldwide, but its prevalence is highest in East Asian countries [5].

MMD has a bimodal age distribution for peak incidence, with peaks in children who are approximately five years of age and adults in their mid-forties [1,3,6,7,8]. Most children MMD patients develop transient ischemic attacks or cerebral infarctions [9], whereas adult MMD patients are more likely to have a hemorrhagic stroke [8,10,11]. This suggests some variant or impairment of the genetic sequence in the same disease. The majority of MMD cases are sporadic, even though familial MMD cases account for approximately 9–15% of all cases [12,13]. Genetic associations with loci on chromosome 3, 6, 8, 10, and 17 and a specific human leukocyte antigen (HLA) haplotype have been reported [14,15,16,17,18,19,20], but questions about genetic penetrations remain.

*RNF213*, which is located on chromosome 17q25, has been recognized as the major susceptibility gene for MMD in East Asians [21,22], as well as Caucasians and East/South Asians [22,23,24,25]. An association of the p.R4810K polymorphism with intracranial major artery stenosis/occlusion has been reported in Japanese and Korea populations [26,27,28,29]. However, this genetic variant associated with MMD was also observed in patients with non-MMD intracranial stenosis [26,28,29]. The association between *RNF213* and clinical manifestations of MMD in young children and adults remain uncertain.

The *RNF213* variant p.R4810K (c.14429G>A, rs112735431) was first reported to have a high level of association with MMD on a large scale [30]. The *RNF213* variants R4859K [21] and R4810K [22] correspond to rs112735431, while R4859K is based on the computer predicted open-reading frame in the database [21].

In the present study, four single nucleotide polymorphisms (SNPs) in exon region were identified through previous studies [21,22,29] and a database search: *RNF213* 4448G>A, *RNF213* 4810G>A, *RNF213* 4863G>A, and *RNF213* 4950G>A. These four SNPs were selected on the basis of information in the HapMap database (http://hapmap.ncbi.nlm.nih.gov/). Currently, no study has addressed the frequency of *RNF213* variants (4448, 4810, 4863, 4950) in a Korean population with a high prevalence of MMD. Therefore, we have investigated the frequency of MMD-related *RNF213* variants in a cohort of Korean patients with MMD compared with healthy controls.

## 2. Results

### 2.1. Genetic Analysis

Table 1 shows the demographic characteristics for patients with MMD in the present study. A comparison of genotype frequencies between moyamoya patients and control subjects of the *RNF213* 4448G>A, *RNF213* 4810G>A, *RNF213* 4863G>A, and *RNF213* 4950G>A polymorphisms is shown in Table 2. There were statistically significant differences between moyamoya patients and controls in *RNF213* 4810G>A and *RNF213* 4950G>A (Table 2).

In subgroup analyses (Table 3 and Table 4), the GA genotype of the *RNF213* 4810GA was more frequent in moyamoya disease (*p* < 0.001; GG vs. AA) and the comparison with the GG+AA genotype was also significantly different in moyamoya patients. The *RNF213* 4950G>A genotype was more frequent in moyamoya disease (*p* = 0.008, GG vs. AA), with high frequencies of the *RNF213* 4950G>A polymorphisms in MMD.

We conducted subgroup analyses of the *RNF213* polymorphisms (Table 3 and Table 4). *RNF213* 4950G>A was more frequent in the ischemic type than in the hemorrhagic type. *RNF213* 4810G>A was more frequent in both the ischemic and hemorrhagic types of MMD.

In the pediatric group (<18 years of age), *RNF213* 4810G>A was more frequent than in the control group *(p* < 0.001). In the adult MMD group, *RNF213* 4810G>A *(p* = 0.004) and *RNF213* 4950G>A were more frequent (*p =* 0.006).

### 2.2. Haplotype Analysis

Haplotype analysis was conducted, as shown in Table 5 and Table 6. The G-G-A-G (*p* = 0.009), G-A-G-G (*p* < 0.001), G-A-G-A (*p* < 0.001), and G-A-A-G (*p* < 0.001) (*RNF213* 4448/4810/4863/4950) haplotypes were significantly higher in moyamoya patients, while the G-G-A-G haplotype (*RNF213* 4448/4810/4863/4950) was lower in MMD patients compared to the group. The G-G-A and G-A-G haplotypes (*RNF213* 4448/4810/4950) were significantly higher in MMD patients, and the A-G-G (*RNF213 4*810/4863/4950) haplotype was more frequent in MMD patients. In addition, we performed haplotype analysis by dividing the MMD patients into two groups: the pediatric group (<18 years old) and the adult group (≥18 years old) (Tables S1–S4). Interestingly, the G-G-A-G (*p* = 0.005), G-A-G-G (*p* < 0.0001), and G-A-A-G (*p* = 0.033) (*RNF213* 4448/4810/4863/4950) haplotypes were associated with moyamoya disease risk in the pediatric group whereas G-A-G-A (*p* < 0.0001) elevated moyamoya disease risk in the adult group. The genotype combination frequency of *RNF213* in MMD patients and control subjects is shown in Table 7. GG/GA (*RNF213* 4448G>A/4810G>A) was more frequent in the MMD group. GA/GG *(RNF213* 4810G>A/4863G>A) and GA/GA (*RNF213* 4810G>A/4863G>A) were more frequent in the MMD group. GA/GG and GA/GA *(RNF213* 4810G>A/4950G>A) were more frequent in the MMD group.

### 2.3. Screening with World Population for RNF213 Polymorphisms

Table 8 shows the major and minor allele frequencies of the *RNF213* polymorphisms in different world populations according to the 1000 Genome Project database (http://www.internationalgenome.org/). Our study detected *RNF213* 4448G>A, 4810G>A, 4863G>A, and 4950G>A allele frequencies in control subjects and MMD patients.

## 3. Discussion

At least 24 genetic changes in the *RNF213* gene have been associated with moyamoya disease [31,32]. Three individual studies of MMD patients have revealed high frequencies of the same single base substitution (nonsynonymous mutation) as well as the c.14576G>A (p.R4859K) variant in *RFP213* (a gene located in chromosome 17q) [21,22,23,24,25,26,27,28,29,30,31,32,33]. The c.14576G>A in *RNF213* is present in ~2% of East Asian populations, which is a relatively higher rate compared with Caucasians [21,22,23,24,25,26,27,28,29,30,31,32,33]. The *RNF213* gene can be used as a biomarker to predict prognosis, as it has been reported that the *RNF213* gene correlates with the early-onset and severe forms of MMD [33]. The *RNF213* p.Arg4810Lys variant is reportedly associated with ischemic-type MMD, while *RNF213* non-p.Arg4810Lys variants are associated with hemorrhagic type MMD [23].

The single-nucleotide polymorphism of c.14576G>A, p.R4859K, in *RNF-213* has been detected in 95% of familial cases and 79% of sporadic cases of MMD [21]. Nevertheless, some MMD patients do not carry the c.14576G>A variant and this portion is higher in western countries [34].

Miyawaki et al. found that *RNF213* (14576G>A) was higher in intracranial major artery stenosis/occlusion (ICASO) patients, as in the MMD group [26], and they suggest ICASO without signs of MMD is a genetic variant associated with MMD. Bang et al. suggest that *RNF213* is a susceptibility gene for both MMD and ICAS in East Asians. Further studies are needed on *RNF213* variants in ICASO patients outside East Asian populations [27]. However, two variants of *RNF213* (p.Arg2438Cys and p.Ala2826Thr) were found in intracranial aneurysm patients in a French-Canadian population [35]. Interestingly, our results differed with the results of a previous study [27] that suggested that 4810G>A is the only variant that is strongly associated with MMD in Korean patients. In this study, our results suggested that *RNF213* 4810 and 4950 were associated with MMD risk, furthermore our result found that frequencies of *RNF213* 4448G>A, 4863G>A, and 4950G>A hetero genotype. We think that this difference is due to the difference in how the genotypes were identified. We used the classical method of using polymerase chain reaction-restriction fragment length polymorphism (PCR-RFLP) and confirmed the genotypes of the individual samples, whereas the previous study performed the MALDI-TOF method, which is likely to cause errors because it confirms the genotype of many samples for many variants.

The *RNF213* gene encodes a protein with 5256 amino acids harboring a RING (Really Interesting New Gene) finger motif and an AAA (ATPase associated with a variety of cellular activities) domain, indicating the presence of both E3 ubiquitin ligase activity and an energy-dependent E3 ubiquitin ligase (which has several subtypes), an enzyme that ubiquitinates specific target proteins, resulting in degradation by proteasomes [26]. The *RNF213* variant associated with MMD prevails, but it is also found in other vascular diseases such as cerebrovascular stenosis [26], albeit not in the Caucasian MMD population [36]. In *RNF213*-deficient mice, an abnormal vascular network does not develop at the base of the brain [37]. The *RNF*213 variant is an important SNP that is not specific to MMD alone.

The c.14576G>A variant is mainly detected in Japanese, Korean, and Chinese populations [33,38,39]. However, the frequency in the latter population is much lower than those of the former two populations [34]. *RNF213* exhibits strong and obvious ethnic diversity [32].

The biochemical function and pathological role of *RNF213* have not been completely clarified. Disruption of the RNF213 first B motif disrupts ATP hydrolysis cyclicity, inhibiting angiogenesis, and this reduced anti-angiogenic activity of interferon beta 1 (IFNb) is partially mediated by *RNF213*, which acts as a mediator downstream of the IFNb signaling pathway [32]. The overexpression of *RNF213* R4810K, but not wild type *RNF213*, suggests that *RNF213* R4810K over expression mimics IFNb action. Koizumi et al. suggest that *RNF213* R4810K is the major detrimental factor that elicits endothelial cell dysfunction. Pro-inflammatory signals such as IFNs can activate the transcription of *RNF213* [32]. The model assumes that any of three independent abnormalities, such as endothelial dysfunction, smooth muscle cell dysfunction, and abnormal hemostasis, can exaggerate the proliferation of smooth muscle cells (SMCs) and that each abnormality can result in vascular stenosis [32].

Genome-wide association study (GWAS) approaches are now being applied to MMD with the hope of uncovering the underlying pathogenic mechanisms [40]. A GWAS was recently performed in Japanese MMD patients and demonstrated a strong association of MMD risk with chromosome 17q25-ter [21]. These GWAS studies will need further investigation to solidly replicate the results in modern genetic studies based on familial or non-familial MMD. Subgroup analysis was performed for adult versus pediatric and ischemic versus hemorrhagic groups. The former primarily presents with ischemia, while the latter presents with intracranial hemorrhage [6,41]. The progression of MMD has generally been considered to occur exclusively in childhood, with angiographic characteristics completed before adulthood [42,43]. Kuroda and colleagues reported in a multicenter observational study on adult onset MMD that the incidence of the disease progression in adult cases was not as rare as originally considered [44]. Miyatake et al. [33] reported that patients with childhood-onset MMD and the homozygous polymorphism of c.14576G>A in *RNF213* were more likely to have an earlier onset and more severe mortality because of the rapid progression of vascular stenosis. Han et al. suggested that *RNF213* R4810K is associated with the ischemic type , and A4399T is associated with the hemorrhagic type [23]. A genetic animal *RNF213* knock-in model has been debated, however, the *RNF213* genetic animal model failed to mimic the MMD model. Liu reported that *RNF213* knock-down zebra fish have abnormal surrounding vessels [22]. Sonobe et al. [37] did not observe any modification of angiogenesis after they generated mice that lack *RNF213*. *RN 213* knock out (KO) animal models have yielded conflicting results in the cerebrum and hind limbs [45]. Fujimura et al. speculated that *RNF213* influences vascular remodeling in chronic ischemia [38]. Allele frequencies of *RNF213* polymorphisms (4448G>A, 4810G>A, 4863G>A, and 4950G>A) in different world populations are presented in Table 8.

SNP studies have some limitations. First, a question remains about how the p.R4810K variant or nine other variants impair the physiological function of *RNF213*, resulting in moyamoya disease [22]. The second limitation is a lack of a pathologic process for MMD development from *RNF213*. Third, large population-based case-control analyses or analyses centered on family-based designs are needed. However, SNP studies have many advantages over other genetic studies, the benefits of which depend on how SNPs will be exploited in relevant study designs and what traits and diseases will be the focus of these studies [46].

We have considered some of the unique aspects of SNPs and their relative advantages and disadvantages in human population-based analyses [46]. We believe that wide-scale progress in genetically identifying MMD is needed because MMD appears to be a multifactorial, polygenic spectrum disorder.

## 4. Materials and Methods

### 4.1. Subjects

One hundred seventeen consecutive Korean patients with moyamoya disease (mean age, 23.20 ± 17.75 years; 77 females (65.8%), 40 males (34.2%)) were recruited for this study. MMD patients were diagnosed and enrolled based on the presence of clinical ischemic or hemorrhagic symptoms in combination with vascular lesions in magnetic resonance imaging (MRI) or magnetic resonance angiography (MRA) [47].

The control group was comprised of 253 healthy subjects (mean age 25.60 ± 16.98 years; 145 females (57.3%); 108 males (42.7%)) from the same regional background as the MMD patients. We recruited the age- and sex-matched subjects from outpatient clinics at Severance Hospital, CHA Bundang Medical Center, Chungbuk National University Hospital (Cheongju, Korea). Table 1 shows demographic characteristics of patients with the disease and control subjects. We divided the MMD patients into pediatric (<18 years) and adult (≥18 years) groups. We further divided the moyamoya patients into ischemic or hemorrhagic groups based on clinical and MRI findings.

All participants gave informed written consent prior to enrollment in the study. The institutional review boards of Severance Hospital (4-2008-0308), CHA Bundang Medical Center (PBC09-103), and Chungbuk National University Hospital (2014-08-010-005) approved this study.

### 4.2. RNF213 Genotyping

DNA was extracted from leukocytes using a G-DEX II Genomic DNA Extraction kit (Intron Biotechnology, Seongnam, Korea) according to the manufacturer’s instructions. To analyze *RNF213* genotypes, we chose polymerase chain reaction-restriction fragment length polymorphism (PCR-RFLP).

*RNF213* 4448G>A was detected using a forward primer (5′-TTG CCA ACT AAG CCC TCG AAA CAA-3′) and a reverse primer (5′-CAA CAA TGG CAC AGA ATT GTC-3′). The 230-bp PCR product was then digested with 5U *Alu*I. A digestion product of 230-bp represented the AA genotype; fragments of 230-bp, 135-bp, and 95-bp represented the AG genotype; 135-bp and 95-bp products represented the GG genotype.

The *RNF213* 4810G>A polymorphism was detected by PCR-RFLP analysis using forward (5′-AGC AGA GCT GAG GCT GGT AA-3′) and reverse (5′-CTG TCA GAG CAG AGC CAC AC-3′) primers. The 151-bp product was digested with 3U *Hpy*188I. A restriction fragment of 146-bp and 5-bp represented the AA genotype; fragments of 146-bp, 104-bp, 42-bp, and 5-bp represented the GA genotype; and 104-bp, 42-bp, and 5-bp products represented the GG genotype.

To detect the *RNF213* 4863G>A genotypes, PCR-RFLP analysis was performed with forward (5′-TGT GTG TGG AGC TGA TGG CT-3′) and reverse (5′-AGG GAG GAG ATA CAG ACC AGA CT-3′) primers. The length of the amplified fragment was 867-bp. PCR products were digested with 5U *Hpy*188I. For 4863G>A, restriction products of 867-bp identified the GG genotype; products of 867-bp, 719-bp, and 148-bp represented the GA genotype; and 719-bp and 148-bp products represented the AA genotype.

The *RNF213* 4950G>A polymorphism was detected by PCR-RFLP analysis using forward (5′-GGT GGA GGA GGG CAG AGA GAC CGT GCA CGA-3′) and reverse (5′- CTT CCC TCT CTC GAG AAA CAC ACC AA-3′) primers. The 188-bp product was digested with 3U *Bss*SI. A restriction fragment of 162-bp and 26-bp represented the GG genotype; fragments of 188-bp, 162-bp, and 26-bp represented the GA genotype; and 188-bp products represented the AA genotype. The *RNF213* 4448G>A, 4810G>A, 4863G>A, and 4950G>A polymorphisms were digested by *Alu*I, *Hpy*188I, *Hpy*188I, and *Bss*SI, respectively, for 16 h at 37 °C (New England BioLabs, Beverly, MA, USA). The PCR annealing temperature was 64 °C for all polymorphisms, with 35 amplification cycles for *RNF213* polymorphisms. The reaction product (12 μL) was run on a 3.0% ethidium bromide-stained agarose gel and confirmed under ultraviolet illumination.

**Figure 1:**
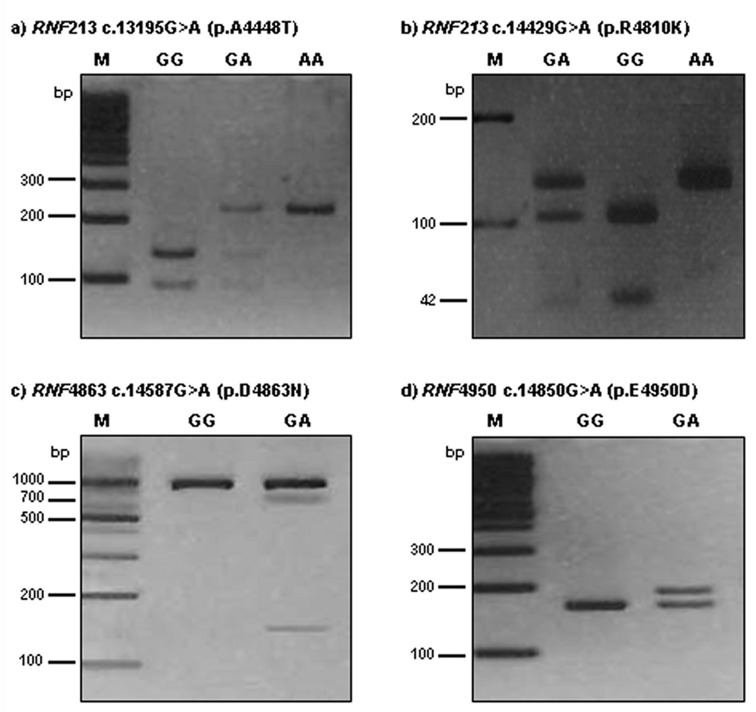
Polymorphism analysis of *RNF213* 4448, 4810, 4863, and 4950 in moyamoya disease patients. Polymorphisms analysis of *RNF213* genes amplicon by agarose gel electrophoresis after restriction endonuclease digestion. (**a**) *RNF213* 4448 c.13195G>A site was digested by *Alu*I resulting in the appearance of the GG (wild type, 135/95 bp), GA (heterozygous type, 230/135/95 bp), and AA (mutant type, 230 bp) genotypes in moyamoya disease patients; (**b**) *RNF213 4810* c.14429G>A site was digested by *Hpy*188I resulting in the appearance of the GG (wild type, 104/42/5 bp), GA (heterozygous type, 146/104/42/5bp), and AA (mutant type, 146 bp) genotypes in moyamoya disease patients. DNA fragments that were 5 bp or less were too small to be seen on 3% agarose gel; (**c**) *RNF4863* c.14587G>A site was digested by *Hpy*188I resulting in the appearance of the GG (wild type, 867 bp) and GA (hetero type, 867/719/148 bp) genotypes in moyamoya disease patients. No mutant type (AA) was found in this study. (**d**) *RNF4950* c.14850G>A site was digested by *Bss*SαI resulting in the appearance of the GG (wild type, 162/26 bp) and GA (heterozygous type, 188/162/26 bp) genotypes. The mutant type (AA) was not found in results of restriction fragment length polymorphism (RFLP). DNA fragments that were 26 bp or less were too small to be seen on 3% agarose gel.

We randomly repeated approximately 10% of the PCR assays for each of the miRNA polymorphisms and checked the results for concordance by DNA sequencing using an automatic sequencer (ABI3730x1 DNA analyzer; Applied Biosystems, Foster City, CA, USA). The concordance of the quality control samples was 100%. Polymorphism analysis was performed for *RNF213 4*448, 4810, 4863, and 4950 in moyamoya disease patients. Polymorphism analysis of *RNF213* gene amplicons was performed by agarose gel electrophoresis after restriction endonuclease digestion (Figure 1).

In this study, we investigated exome analysis and identified ring finger protein 213 (*RNF213*; DDBJ/EMBL/GenBank accession number AB537889) (National Center for Biotechnology Information (NCBI) in Table S5). Questions remain regarding how the p.R4810K variant or nine other variants impair RNF213 protein, thereby resulting in MMD.

### 4.3. Statistical Analyses

The genotype frequencies for *RNF213* polymorphism were compared by Hardy-Weinberg equilibrium (HWE) test [48]. To analyze the demographic characteristics of moyamoya disease, we used the Mann–Whitney and chi-square (χ^2^) tests for continuous and categorical data, respectively. The relationships between *RNF213* and MMD patients (pediatric or adult) were calculated according to the odds ratios (ORs) and 95% confidence intervals (CIs) using Fisher’s exact test. The adjusted odds ratios (AORs) for four polymorphisms of the *RNF213* gene were calculated using multiple logistic regression analyses with gender and age. We considered Hardy-Weinberg equilibrium in the genotype distribution. We used GraphPad Prism 4.0 (GraphPad Software, Inc., San Diego, CA, USA) and StatsDirect software (version 2.4.4; StatsDirect Ltd., Altrincham, UK) to perform statistical analyses. We used HAPSTAT (version 3.0; University of North Carolina, Chapel Hill, NC, USA) and Haploview 4.2 (Broad Institute, Cambridge, MA, USA) to perform Haplotype analyses. The adjusted ORs and 95% CIs were calculated by statistical software (version 2.4.4; StatsDirect Ltd., Altrincham, UK).

## 5. Conclusions

In summary, *RNF213* 4810G>A and *RNF213* 4950G>A were more frequent in MMD patient. Current *RNF213* SNP studies suggest that *RNF213* 4810G>A and 4950G>A are strongly associated with Korean MMD, both in pediatric or adult patients as well as in the ischemic or hemorrhagic types. Our results show that polymorphism of *RNF213* 4810G>A and 4950G>A are associated with the occurrence of moyamoya disease, and that 4810G>A may affect the general moyamoya prevalence, while 4950G>A is particularly relevant to the occurrence of moyamoya in the adult group. It is therefore thought that it can serve as a potential biomarker of moyamoya disease in adult groups.

## Figures and Tables

**Table 1:** Demographic characteristics of moyamoya disease patients and subjects.

Characteristics	Controls (*n* = 253)	Cases (*n* = 117)	*p*
Age (mean ± S.D.)	25.60 ± 16.98	23.20 ± 17.75	0.213
Age ≥ 18	37.69 ± 10.40 (151)	37.70 ± 12.81 (60)	0.995
Age < 18	7.71 ± 4.05 (102)	7.93 ± 4.11 (57)	0.740
Sex (F:M)	145:108	77:40	0.151
Clinical findings	-		
Transient ischemic attack	-	52	
Cerebral infarction	-	50	
Cerebral hemorrhage	-	17	
Surgery	-	65	
Suzuki stage	-		
Right	-	3.17 ± 0.78	
Left	-	3.89 ± 1.27	

*p*-Values were calculated by a two-sided *t*-test for continuous variables and a χ-square test for categorical data. Abbreviation: S.D., standard deviation. F, female; M, male.

**Table 2:** Genotype frequencies of *RNF213* in moyamoya disease (MMD) patients and control subjects.

Genotype	Controls, *n* = 253 (%)	MMD, *n* = 117 (%)	COR (95% CI)	*p*	FDR-*P*	AOR (95% CI) *	*p*	FDR-*P*
*RNF213* 4448G>A								
GG	219 (86.6)	109 (93.2)	1.000 (reference)			1.000 (reference)		
GA	33 (13.0)	8 (6.8)	0.487 (0.218–1.090)	0.080	0.107	0.472 (0.210–1.061)	0.069	0.092
AA	1 (0.4)	0	N/A	N/A		N/A	N/A	
Dominant (GG vs. GA+AA)			0.473 (0.212–1.056)	0.068	0.091	0.463 (0.207–1.039)	0.062	0.083
HWE *P*	0.837	0.702						
*RNF213* 4810G>A								
GG	234 (92.5)	14 (12.0)	1.000 (reference)			1.000 (reference)		
GA	19 (7.5)	102 (87.2)	89.73 (43.31–185.9)	<0.001	0.004	94.43 (44.45–200.6)	<0.001	0.004
AA	0	1 (0.9)	N/A	N/A		N/A	N/A	
Dominant (GG vs. GA+AA)			90.61 (43.74–187.7)	<0.001	0.004	95.34 (45.45–192.8)	<0.001	0.004
HWE *P*	0.535	0						
*RNF213* 4863G>A								
GG	223 (88.1)	108 (92.3)	1.000 (reference)			1.000 (reference)		
GA	30 (11.9)	9 (7.7)	0.619 (0.284–1.351)	0.229	0.229	0.574 (0.258–1.278)	0.174	0.174
AA	0	0	N/A	N/A		N/A	N/A	
Dominant (GG vs. GA+AA)			0.619 (0.284–1.351)	0.229	0.229	0.574 (0.258–1.278)	0.174	0.174
HWE *P*	0.316	0.665						
*RNF213* 4950G>A								
GG	224 (88.5)	91 (77.8)	1.000 (reference)			1.000 (reference)		
GA	29 (11.5)	26 (22.2)	2.207 (1.232–3.952)	0.008	0.016	2.194 (1.216–3.958)	0.009	0.018
AA	0	0	N/A	N/A		N/A	N/A	
Dominant (GG vs. GA+AA)			2.207 (1.232–3.952)	0.008	0.016	2.194 (1.216–3.958)	0.009	0.018
HWE *P*	0.334	0.176						

* Adjusted by age and gender. MMD, moyamoya disease. Abbreviation: MMD, moyamoya disease; COR, crude odd ratio; CI, confidence interval, FDR, false discovery rate; AOR, adjusted odd ratio; HWE, Hardy-Weinberg equilibrium; N/A, not application.

**Table 3:** Genotype frequencies of *RNF213* in moyamoya patient subtypes and control subjects.

Genotype	Controls, *n* = 253 (%)	Ischemic Moyamoya, *n* = 100 (%)	AOR (95% CI) *	*p*	FDR-*P*	Hemorrhagic Moyamoya, *n* = 17 (%)	AOR (95% CI) *	*p*	FDR-*P*
*RNF213* 4448G>A									
GG	219 (86.6)	93 (93.0)	1.000 (reference)			16 (94.1)	1.000 (reference)		
GA	33 (13.0)	7 (7.0)	0.479 (0.204–1.129)	0.093	0.124	1 (5.9)	0.433 (0.055–3.429)	0.428	0.428
AA	1 (0.4)	0	N/A	N/A		0	N/A	N/A	
Dominant (GG vs. GA+AA)			0.472 (0.201–1.110)	0.085	0.113		0.415 (0.053–3.278)	0.404	0.404
*RNF213* 4810G>A									
GG	234 (92.5)	10 (10.0)	1.000 (reference)			4 (23.5)	1.000 (reference)		
GA	19 (7.5)	89 (89.0)	111.8 (49.07–254.8)	<0.001	0.004	13 (76.5)	74.51 (16.57–335.0)	<0.001	0.003
AA	0	1 (1.0)	N/A	N/A		0	N/A	N/A	
Dominant (GG vs. GA+AA)			113.0 (49.60–257.4)	<0.001	0.004		74.51 (16.57–335.0)	<0.001	0.003
*RNF213* 4863G>A									
GG	223 (88.1)	91 (91.0)	1.000 (reference)			17 (100.0)	1.000 (reference)		
GA	30 (11.9)	9 (9.0)	0.632 (0.282–1.414)	0.264	0.264	0	N/A	N/A	
AA	0	0	N/A	N/A		0	N/A	N/A	
Dominant (GG vs. GA+AA)			0.632 (0.282–1.414)	0.264	0.264		N/A	N/A	
*RNF213* 4950G>A									
GG	224 (88.5)	78 (78.0)	1.000 (reference)			13 (76.5)	1.000 (reference)		
GA	29 (11.5)	22 (22.0)	2.234 (1.199–4.163)	0.011	0.022	4 (23.5)	2.168 (0.649–7.244)	0.209	0.314
AA	0	0	N/A	N/A		0	N/A	N/A	
Dominant (GG vs. GA+AA)			2.234 (1.19–4.163)	0.011	0.022		2.168 (0.649–7.244)	0.209	0.314

* Adjusted by age and gender. MMD, moyamoya disease. Abbreviation: AOR, adjusted odd ratio; CI, confidence interval, FDR, false discovery rate; N/A, not application.

**Table 4:** The frequencies of the *RNF213* polymorphisms according to age.

Genotype	Age < 18 Years					Age ≥ 18 Years				
	Controls, *n* = 102 (%)	Moyamoya, *n* = 57 (%)	AOR (95% CI) *	*p*	FDR-*P*	Controls, *n* = 151 (%)	Moyamoya, *n* = 60 (%)	AOR (95% CI) *	*p*	FDR-*P*
*RNF213* 4448G>A										
GG	89 (87.3)	51 (89.5)	1.000 (reference)			130 (86.1)	58 (96.7)	1.000 (reference)		
GA	13 (12.7)	6 (10.5)	0.800 (0.284–2.251)	0.672	0.672	20 (13.2)	2 (3.3)	0.223 (0.050−0.986)	0.048	0.064
AA	0	0	N/A	N/A		1 (0.7)	0	N/A	N/A	
Dominant (GG vs. GA+AA)			0.800 (0.284−2.251)	0.672	0.672			0.214 (0.048−0.944)	0.042	0.056
*RNF213* 4810G>A										
GG	89 (87.3)	7 (12.3)	1.000 (reference)					1.000 (reference)		
GA	13 (12.7)	49 (86.0)	48.99 (18.00−133.3)	<0.001	0.004	145 (96.0)	7 (11.7)	199.6 (60.95−653.6)	<0.001	0.004
AA	0	1 (1.8)	N/A	N/A		6 (4.0)	53 (88.3)	N/A	N/A	
Dominant (GG vs. GA+AA)			50.06 (18.43−136.0)	<0.001	0.004	0	0	199.6 (60.95−653.6)	<0.001	0.004
*RNF213* 4863G>A										
GG	78 (76.5)	51 (89.5)	1.000 (reference)			145 (96.0)	57 (95.0)	1.000 (reference)		
GA	24 (23.5)	6 (10.5)	0.395 (0.150−1.039)	0.060	0.120	6 (4.0)	3 (5.0)	1.340 (0.321−5.592)	0.688	0.688
AA	0	0	N/A	N/A		0	0	N/A	N/A	
Dominant (GG vs. GA+AA)			0.395 (0.150−1.039)	0.060	0.120			1.340 (0.321−−5.592)	0.688	0.688
*RNF213* 4950G>A										
GG	91 (89.2)	49 (86.0)	1.000 (reference)			133 (88.1)	42 (70.0)	1.000 (reference)		
GA	11 (10.8)	8 (14.0)	1.260 (0.470−3.379)	0.646	0.672	18 (11.9)	18 (30.0)	3.096 (1.473−6.506)	0.003	0.006
AA	0	0	N/A	N/A		0	0	N/A	N/A	
Dominant (GG vs. GA+AA)			1.260 (0.470−3.379)	0.646	0.672			3.096 (1.473−6.506)	0.003	0.006

* Adjusted by age and gender. MMD, moyamoya disease. Abbreviation: AOR, adjusted odd ratio; CI, confidence interval, FDR, false discovery rate; N/A, not application.

**Table 5:** Haplotype analysis of *RNF213* in MMD patients and control subjects.

Haplotypes	Controls (2*n* = 506)	MMD (2*n* = 234)	OR (95% CI)	*p*	FDR-*P*
*RNF213* 4448G>A/4810G>A/4863G>A/4950G>A					
G-G-G-G	0.7932 (401)	0.4795 (112)	1.000 (reference)		
G-G-G-A	0.0534 (27)	0.0610 (14)	1.856 (0.942–3.660)	0.081	0.122
G-G-A-G	0.0488 (25)	0	0.070 (0.004–1.159)	0.004	0.009
G-G-A-A	0	0	N/A	N/A	
G-A-G-G	0.0226 (11)	0.3421 (80)	26.04 (13.40–50.60)	<0.001	0.003
G-A-G-A	0.0024 (1)	0.0447 (10)	35.80 (4.532–282.8)	<0.001	0.003
G-A-A-G	0.0105 (5)	0.0385 (9)	6.445 (2.117-19.62)	0.001	0.003
G-A-A-A	0	0	N/A	N/A	
A-G-G-G	0.0656 (33)	0.015 (4)	0.434 (0.151–1.251)	0.144	0.185
A-G-G-A	0.0015 (1)	0	1.190 (0.048–29.43)	1.000	1.000
A-G-A-G	0	0	N/A	N/A	
A-G-A-A	0	0	N/A	N/A	
A-A-G-G	0.0021 (1)	0.0137 (3)	10.74 (1.106–104.3)	0.036	0.065
A-A-G-A	0	0.0054 (1)	10.71 (0.433–264.8)	0.220	0.248
A-A-A-G	0	0	N/A	N/A	
A-A-A-A	0	0	N/A	N/A	
*RNF213* 4448G>A/4810G>A/4863G>A					
G-G-G	0.8464 (428)	0.5405 (126)	1.000 (reference)		
G-G-A	0.0488 (25)	0	0.066 (0.004–1.099)	0.003	0.005
G-A-G	0.0252 (13)	0.3863 (91)	23.78 (12.86–43.95)	<0.001	0.003
G-A-A	0.0104 (5)	0.0385 (9)	6.114 (2.012–18.58)	0.001	0.003
A-G-G	0.0671 (34)	0.0150 (4)	0.400 (0.139–1.148)	0.103	0.103
A-G-A	0	0	N/A	N/A	
A-A-G	0.0020 (1)	0.0192 (4)	13.59 (1.504–122.7)	0.012	0.015
A-A-A	0	0	N/A	N/A	
*RNF213* 4448G>A/4810G>A/4950G>A					
G-G-G	0.8406 (425)	0.4333 (101)	1.000 (reference)		
G-G-A	0.0539 (27)	0.1054 (25)	4.373 (2.468–7.748)	<0.001	0.004
G-A-G	0.0346 (18)	0.4271 (100)	27.62 (16.18–47.15)	<0.001	0.004
G-A-A	0.0017 (1)	0	1.742 (0.070–43.10)	1.000	1.000
A-G-G	0.0663 (34)	0.0168 (4)	0.552 (0.193–1.582)	0.278	0.389
A-G-A	0.0017 (1)	0	1.742 (0.070–43.10)	1.000	1.000
A-A-G	0.0012 (1)	0.0117 (3)	15.74 (1.620–152.9)	0.025	0.058
A-A-A	0	0.0057 (1)	15.68 (0.634–387.9)	0.194	0.400
*RNF213* 4448G>A/4863G>A/4950G>A					
G-G-G	0.8155 (413)	0.8210 (192)	1.000 (reference)		
G-G-A	0.0561 (28)	0.1064 (25)	1.921 (1.091–3.382)	0.032	0.110
G-A-G	0.0593 (30)	0.0385 (9)	0.645 (0.300–1.386)	0.290	0.387
G-A-A	0	0	N/A	N/A	
A-G-G	0.0679 (34)	0.0295 (7)	0.443 (0.193–1.017)	0.055	0.110
A-G-A	0.0013 (1)	0.0047 (1)	2.151 (0.134–34.60)	0.535	0.535
A-A-G	0	0	N/A	N/A	
A-A-A	0	0	N/A	N/A	
*RNF213* 4810G>A/4863G>A/4950G>A					
G-G-G	0.8587 (435)	0.4947 (116)	1.000 (reference)		
G-G-A	0.0549 (28)	0.0608 (14)	1.875 (0.956–3.677)	0.080	0.080
G-A-G	0.0488 (25)	0	0.073 (0.004–1.214)	0.004	0.005
G-A-A	0	0	N/A	N/A	
A-G-G	0.0247 (12)	0.3557 (83)	25.94 (13.69–49.15)	<0.001	0.002
A-G-A	0.0024 (1)	0.0503 (12)	45.00 (5.789–349.8)	<0.001	0.002
A-A-G	0.0105 (5)	0.0385 (9)	6.750 (2.219–20.53)	0.001	0.002
A-A-A	0	0	N/A	N/A	

Abbreviation: MMD, moyamoya disease; OR, odd ratio; CI, confidence interval, FDR, false discovery rate; N/A, not application.

**Table 6:** Haplotype analysis of *RNF213* in MMD patients and control subjects.

Haplotype	Controls (2*n* = 506)	MMD (2*n* = 234)	OR (95% CI)	*p*	FDR-*P*
*RNF213* 4448G>A/4810G>A					
G-G	0.8945 (453)	0.5327 (125)	1.000 (reference)		
G-A	0.0364 (18)	0.4331 (101)	19.53 (11.38–33.49)	<0.001	0.003
A-G	0.0680 (34)	0.0229 (5)	0.512 (0.196–1.336)	0.227	0.227
A-A	0.0012 (1)	0.0113 (3)	10.44 (1.076–101.3)	0.038	0.057
*RNF213* 4448G>A/4863G>A					
G-G	0.8715 (441)	0.9274 (217)	1.000 (reference)		
G-A	0.0593 (30)	0.0385 (9)	0.610 (0.284–1.307)	0.222	0.222
A-G	0.0629 (35)	0.0342 (8)	0.465 (0.212–1.019)	0.063	0.126
A-A	0	0	N/A	N/A	
*RNF213* 4448G>A/4950G>A					
G-G	0.8751 (443)	0.8596 (201)	1.000 (reference)		
G-A	0.0557 (28)	0.1062 (25)	1.968 (1.119–3.461)	0.022	0.066
A-G	0.0675 (34)	0.0293 (7)	0.454 (0.198–1.041)	0.078	0.117
A-A	0.0016 (1)	0.0049 (1)	2.204 (0.137–35.44)	0.528	0.528
*RNF213* 4810G>A/4863G>A					
G-G	0.9136 (462)	0.5556 (130)	1.000 (reference)		
G-A	0.0489 (25)	0	0.069 (0.004–1.150)	0.004	0.004
A-G	0.0271 (14)	0.4060 (95)	24.12 (13.31–43.68)	<0.001	0.002
A-A	0.0104 (5)	0.0385 (9)	6.397 (2.107–19.42)	<0.001	0.002
*RNF213* 4810G>A/4950G>A					
G-G	0.9069 (459)	0.4444 (104)	1.000 (reference)		
G-A	0.0556 (28)	0.1111 (26)	4.098 (2.307–7.282	<0.001	0.002
A-G	0.0358 (18)	0.4444 (104)	25.50 (14.80–43.93)	<0.001	0.002
A-A	0.0018 (1)	0	1.466 (0.059–36.26)	1.000	1.000
*RNF213* 4863G>A/4950G>A					
G-G	0.8834 (447)	0.8504 (199)	1.000 (reference)		
G-A	0.0573 (29)	0.1111 (26)	2.014 (1.156–3.509)	0.016	0.032
A-G	0.0593 (30)	0.0385 (9)	0.674 (0.314–1.446)	0.372	0.372
A-A	0	0	N/A	N/A	

Abbreviation: MMD, moyamoya disease; OR, odd ratio; CI, confidence interval, FDR, false discovery rate; N/A, not application.

**Table 7:** Genotype combination frequency of *RNF213* in MMD patients and control subjects.

Genotype	Controls, *n* = 253 (%)	MMD, *n* = 117 (%)	AOR (95% CI) *	*p*	FDR-*P*
*RNF213* 4448G>A/4810G>A					
GG/GG	202 (79.8)	13 (11.1)	1.000 (reference)		
GG/GA	17 (6.7)	95 (81.2)	93.58 (42.23–207.4)	<0.001	0.002
GG/AA	0	1 (0.9)	N/A	N/A	
GA/GG	31 (12.3)	1 (0.9)	0.483 (0.061–3.845)	0.492	0.492
GA/GA	2 (0.8)	7 (6.0)	51.99 (9.165–294.9)	<0.001	0.002
GA/AA	0	0	N/A	N/A	
AA/GG	1 (0.4)	0	N/A	N/A	
AA/GA	0	0	N/A	N/A	
AA/AA	0	0	N/A	N/A	
*RNF213* 4448G>A/4863G>A					
GG/GG	191 (75.5)	100 (85.5)	1.000 (reference)		
GG/GA	28 (11.1)	9 (7.7)	0.586 (0.260–1.318)	0.196	0.196
GG/AA	0	0	N/A	N/A	
GA/GG	31 (12.3)	8 (6.8)	0.470 (0.207–1.068)	0.072	0.144
GA/GA	2 (0.8)	0	N/A	N/A	
GA/AA	0	0	N/A	N/A	
AA/GG	1 (0.4)	0	N/A	N/A	
AA/GA	0	0	N/A	N/A	
AA/AA	0	0	N/A	N/A	
*RNF213* 4448G>A/4950G>A					
GG/GG	193 (76.3)	85 (72.6)	1.000 (reference)		
GG/GA	26 (10.3)	24 (20.5)	2.101 (1.131–3.903)	0.019	0.057
GG/AA	0	0	N/A	N/A	
GA/GG	30 (11.9)	6 (5.1)	0.451 (0.180–1.127)	0.088	0.132
GA/GA	3 (1.2)	2 (1.7)	1.201 (0.193–7.480)	0.844	0.844
GA/AA	0	0	N/A	N/A	
AA/GG	1 (0.4)	0	N/A	N/A	
AA/GA	0	0	N/A	N/A	
AA/AA	0	0	N/A	N/A	
*RNF213* 4810G>A/4863G>A					
GG/GG	210 (83.0)	14 (12.0)	1.000 (reference)		
GG/GA	24 (9.5)	0	N/A	N/A	
GG/AA	0	0	N/A	N/A	
GA/GG	13 (5.1)	94 (80.3)	107.3 (48.21–238.9)	<0.001	0.001
GA/GA	6 (2.4)	8 (6.8)	18.08 (5.240–62.40)	<0.001	0.001
GA/AA	0	0	N/A	N/A	
AA/GG	0	0	N/A	N/A	
AA/GA	0	1 (0.9)	N/A	N/A	
AA/AA	0	0	N/A	N/A	
*RNF213* 4810G>A/4950G>A					
GG/GG	207 (81.8)	11 (9.4)	1.000 (reference)		
GG/GA	27 (10.7)	3 (2.6)	1.961 (0.504–7.629)	0.331	0.331
GG/AA	0	0	N/A	N/A	
GA/GG	17 (6.7)	79 (67.5)	89.25 (39.26–202.9)	<0.001	0.002
GA/GA	2 (0.8)	23 (19.7)	216.1 (44.62–1047)	<0.001	0.002
GA/AA	0	0	N/A	N/A	
AA/GG	0	1 (0.9)	N/A	N/A	
AA/GA	0	0	N/A	N/A	
AA/AA	0	0	N/A	N/A	
*RNF213* 4863G>A/4950G>A					
GG/GG	195 (77.1)	83 (70.9)	1.000 (reference)		
GG/GA	28 (11.1)	25 (21.4)	2.060 (1.125–3.772)	0.019	0.057
GG/AA	0	0	N/A	N/A	
GA/GG	29 (11.5)	8 (6.8)	0.557 (0.238–1.303)	0.177	0.266
GA/GA	1 (0.4)	1 (0.9)	2.883 (0.174–47.67)	0.460	0.460
GA/AA	0	0	N/A	N/A	
AA/GG	0	0	N/A	N/A	
AA/GA	0	0	N/A	N/A	
AA/AA	0	0	N/A	N/A	

* Adjusted by age and gender. Abbreviation: MMD, moyamoya disease; AOR, adjusted odd ratio; CI, confidence interval, FDR, false discovery rate; N/A, not application.

**Table 8:** Allele frequencies of *RNF*213 polymorphisms (4448G>A, 4810G>A, 4863G>A, and 4950G>A) in different world populations.

Population	*N*	*RNF*213 4448G>A (rs148731719)		*RNF*213 4810G>A (rs112735431)		*RNF*213 4863G>A (rs760732823)		*RNF*213 4950G>A (rs371441113)		
		G allele	A allele	G allele	A allele	G allele	A allele	G allele	A allele	Database
African	661	0.9985	0.0015	1.0000	0.0000	-	-	1.0000	0.0000	1000 Genome ^a^
Ad Mixed American	347	0.9915	0.0085	1.0000	0.0000	-	-	1.0000	0.0000	
East Asian	504	0.9315	0.0685	0.9980	0.0020	-	-	0.9960	0.0040	
Chinese Dai in Xishuangbanna, China	93	0.8815	0.1185	1.0000	0.0000	-	-	0.9945	0.0055	
Han Chinese in Beijing, China	103	0.9370	0.0630	1.0000	0.0000	-	-	1.0000	0.0000	
Southern Han Chinese	105	0.9240	0.0760	1.0000	0.0000	-	-	0.9950	0.0050	
Japanese in Tokyo, Japan	104	0.9325	0.0675	0.9905	0.0095	-	-	1.0000	0.0000	
Kinh in Ho Chi Minh City, Vietnam	99	0.9800	0.0200	1.0000	0.0000	-	-	0.9900	0.0100	
European	503	0.9920	0.0080	1.0000	0.0000	-	-	1.0000	0.0000	
South Asia	489	0.9940	0.0060	0.9960	0.0040	-	-	1.0000	0.0000	
African	7650	0.9970	0.0030	1.0000	0.0000	1.0000	0.0000	1.0000	0.0000	gnomAD ^b^
Ad Mixed American	16,791	0.9950	0.0050	1.0000	0.0000	1.0000	0.0000	1.0000	0.0000	
Ashkenazi Jewish	4925	0.9830	0.0170	1.0000	0.0000	1.0000	0.0000	1.0000	0.0000	
East Asian	8619	0.9470	0.0530	0.9970	0.0030	0.9990	0.0010	0.9970	0.0030	
Finnish	10,703	0.9960	0.0040	1.0000	0.0000	1.0000	0.0000	1.0000	0.0000	
Non-Finnish European	55,846	0.9920	0.0080	1.0000	0.0000	1.0000	0.0000	1.0000	0.0000	
South Asia	15,391	0.9960	0.0040	0.9996	0.0004	1.0000	0.0000	0.9996	0.0004	
Other (population not assigned)	2734	0.9890	0.0110	1.0000	0.0000	1.0000	0.0000	1.0000	0.0000	
Korean (controls)	253	0.9310	0.0690	0.9660	0.0340	0.9405	0.0595	0.9425	0.0575	Present study
Korean (MMD)	117	0.9660	0.0340	0.5560	0.4440	0.9615	0.0385	0.8890	0.1110	Present study

^a^ The 1000 Genome Project website: https://www.internationalgenome.org/; we checked these polymorphisms frequencies based on GRCh38. ^b^ The Genome Aggregation Database website: http://gnomad.broadinstitute.org/; Official gnomAD release (version 2.0).

## Supplementary Materials

Supplementary materials can be found at www.mdpi.com/1422-0067/18/11/2477/s1.
