# Supplementary material for: The Role of RNF213 4810G>A and 4950G>A Variants in Patients with Moyamoya Disease in Korea
Source: Int J Mol Sci. 2017 Nov 21;18(11):2477. doi: 10.3390/ijms18112477 (PMC5713443; doi:10.3390/ijms18112477)
Supplement: Supplementary file 1 [file ijms-18-02477-s001.pdf]

Supplementary Tables

Table S1. Haplotype analysis (4, 3 site) of *RNF213* in MMD patients and control subjects of pediatric group.

Haplotypes	Controls (2*n* = 204)	Age < 18 Years (2*n* = 114)	OR (95% CI)	*p*	FDR-*P*
RNF213 4448G>A/4810G>A/4863G>A/4950G>A					
G-G-G-G	0.7428 (152)	0.4956 (56)	1.000 (reference)		
G-G-G-A	0.0386 (8)	0.0261 (3)	1.018 (0.261-3.974)	1.000	1.000
G-G-A-G	0.0938 (19)	0.0000 (0)	0.070 (0.004-1.166)	0.005	0.024
G-A-G-G	0.0325 (7)	0.3444 (39)	15.12 (6.392-35.78)	<0.0001	0.001
G-A-G-A	0.0081 (2)	0.0286 (3)	4.071 (0.663-25.02)	0.132	0.263
G-A-A-G	0.0204 (4)	0.0526 (6)	4.071 (1.107-14.97)	0.033	0.111
A-G-G-G	0.0504 (10)	0.0309 (4)	1.086 (0.327-3.603)	1.000	1.000
A-G-G-A	0.0072 (1)	0.0000 (0)	0.900 (0.036-22.43)	1.000	1.000
A-G-A-G	0.0034 (1)	0.0000 (0)	0.900 (0.036-22.43)	1.000	1.000
A-A-G-G	0.0027 (1)	0.0063 (1)	2.714 (0.167-44.16)	0.470	0.784
A-A-G-A	0.0000 (0)	0.0154 (2)	13.50 (0.638-285.7)	0.075	0.188
RNF213 4448G>A/4810G>A/4863G>A					
G-G-G	0.7782 (159)	0.5219 (59)	1.000 (reference)		
G-G-A	0.0960 (20)	0.0000 (0)	0.065 (0.004–1.099)	0.005	0.012
G-A-G	0.0429 (9)	0.3728 (42)	12.58 (5.767–27.43)	<0.0001	0.001
G-A-A	0.0192 (4)	0.0526 (6)	4.042 (1.101–14.84)	0.034	0.057
A-G-G	0.0597 (12)	0.0307 (3)	0.674 (0.183–2.473)	0.765	0.765
A-G-A	0.0024 (0)	0.0000 (0)	N/A	-	
A-A-G	0.0016 (0)	0.0219 (2)	13.40 (0.634–283.5)	0.076	0.095
RNF213 4448G>A/4810G>A/4950G>A					
G-G-G	0.8342 (170)	0.4649 (53)	1.000 (reference)		
G-G-A	0.0396 (8)	0.0526 (6)	2.406 (0.799–7.247)	0.119	0.238
G-A-G	0.0553 (11)	0.4298 (49)	14.29 (6.933–29.45)	<0.0001	0.001
G-A-A	0.0072 (1)	0.0000 (0)	1.062 (0.042–26.48)	1.000	1.000
A-G-G	0.0553 (11)	0.0351 (4)	1.166 (0.356–3.817)	0.760	1.000
A-G-A	0.0072 (1)	0.0000 (0)	1.062 (0.042–26.48)	1.000	1.000
A-A-G	0.0012 (0)	0.0000 (0)	-	-	
A-A-A	0.0000 (0)	0.0175 (2)	15.93 (0.7527–337.3)	0.059	0.177
RNF213 4448G>A/4863G>A/4950G>A					
G-G-G	0.7724 (158)	0.8396 (96)	1.000 (reference)		
G-G-A	0.0476 (10)	0.0551 (6)	0.988 (0.348–2.804)	1.000	1.000
G-A-G	0.1163 (24)	0.0526 (6)	0.412 (0.162–1.043)	0.069	0.278
A-G-G	0.0560 (11)	0.0376 (4)	0.599 (0.185–1.933)	0.584	0.778
A-G-A	0.0064 (1)	0.0151 (2)	3.292 (0.294–36.81)	0.560	0.778
A-A-G	0.0014 (0)	0.0000 (0)	-	-	
RNF213 4810G>A/4863G>A/4950G>A					
G-G-G	0.7925 (162)	0.5263 (60)	1.000 (reference)		
G-G-A	0.0462 (9)	0.0263 (3)	0.900 (0.236–3.438)	1.000	1.000
G-A-G	0.0976 (20)	0.0000 (0)	0.066 (0.004–1.101)	0.005	0.013
A-G-G	0.0359 (7)	0.3509 (40)	15.43 (6.554–36.32)	<0.0001	0.001
A-G-A	0.0077 (2)	0.0439 (5)	6.750 (1.275–35.74)	0.021	0.035
A-A-G	0.0201 (4)	0.0526 (6)	4.050 (1.104–14.86)	0.034	0.042

MMD, moyamoya disease; N/A, not application; FDR, false discovery rate.

Table S2. Haplotype analysis (4, 3site) of *RNF213* in MMD patients and control subjects of adult group.

Haplotypes	Controls (2*n* = 302)	Age ≥ 18 Years (2*n* = 120)	OR (95% CI)	*p*	FDR-*P*
RNF213 4448G>A/4810G>A/4863G>/4950G>A					
G-G-G-G	0.8352 (252)	0.5145 (62)	1.000 (reference)		
G-G-G-A	0.0573 (17)	0.0438 (5)	1.195 (0.425–3.367)	0.782	1.000
G-G-A-G	0.0145 (4)	0.0000 (0)	0.449 (0.023–8.454)	1.000	1.000
G-G-A-A	0.0023 (1)	0.0000 (0)	15.12 (6.392–35.78)	<0.0001	0.001
G-A-G-G	0.0148 (4)	0.3006 (36)	1.347 (0.054–33.48)	1.000	1.000
G-A-G-A	0.0000 (0)	0.0994 (12)	101.0 (5.895–173.0)	<0.0001	0.001
G-A-A-G	0.0031 (1)	0.0182 (2)	8.129 (0.725–91.15)	0.105	0.189
G-A-A-A	0.0000 (0)	0.0068 (1)	12.12 (0.488–301.3)	0.200	0.300
A-G-G-G	0.0708 (21)	0.0000 (0)	0.094 (0.006–1.573)	0.019	0.056
A-A-G-G	0.0021 (1)	0.0167 (2)	8.129 (0.725–91.15)	0.105	0.189
RNF213 4448G>A/4810G>A/4863G>A					
G-G-G	0.8926 (270)	0.5583 (67)	1.000 (reference)		
G-G-A	0.0168 (5)	0.0000 (0)	0.364 (0.020–6.674)	0.587	0.587
G-A-G	0.0147 (4)	0.4000 (48)	48.36 (16.84–138.8)	<0.0001	0.001
G-A-A	0.0030 (1)	0.0250 (3)	12.09 (1.237–118.1)	0.029	0.048
A-G-G	0.0707 (21)	0.0000 (0)	0.093 (0.006–1.559)	0.019	0.047
A-A-G	0.0021 (1)	0.0167 (2)	8.06 (0.720–90.27)	0.106	0.133
RNF213 4448G>A/4810G>A/4950G>A					
G-G-G	0.8495 (257)	0.5145 (62)	1.000 (reference)		
G-G-A	0.0596 (18)	0.0438 (5)	1.151 (0.411–3.222)	0.787	0.787
G-A-G	0.0181 (5)	0.3188 (38)	31.50 (11.91–83.35)	<0.0001	0.000
G-A-A	0.0000 (0)	0.1062 (13)	111.2 (6.520–1898)	<0.0001	0.000
A-G-G	0.0710 (21)	0.0000 (0)	0.096 (0.006–1.604)	0.019	0.031
A-A-G	0.0018 (1)	0.0167 (2)	8.290 (0.739–92.95)	0.102	0.127
RNF213 4448G>A/4863G>A/4950G>A					
G-G-G	0.8498 (257)	0.8130 (98)	1.000 (reference)		
G-G-A	0.0575 (17)	0.1453 (17)	2.622 (1.287–5.342)	0.010	0.039
G-A-G	0.0178 (5)	0.0203 (2)	1.049 (0.200–5.499)	1.000	1.000
G-A-A	0.0021 (1)	0.0047 (1)	2.622 (0.162–42.37)	0.478	0.637
A-G-G	0.0728 (22)	0.0167 (2)	0.238 (0.055–1.033)	0.052	0.105
RNF213 4810G>A/4863G>A/4950G>A					
G-G-G	0.9061 (274)	0.5066 (61)	1.000 (reference)		
G-G-A	0.0572 (17)	0.0517 (6)	1.585 (0.600–4.188)	0.404	0.566
G-A-G	0.0144 (4)	0.0000 (0)	0.496 (0.026–9.339)	1.000	1.000
G-A-A	0.0024 (1)	0.0000 (0)	1.488 (0.060–36.99)	1.000	1.000
A-G-G	0.0168 (5)	0.3248 (39)	35.04 (13.26–92.58)	<0.0001	0.000
A-G-A	0.0000 (0)	0.0918 (11)	102.7 (5.964–1767)	<0.0001	0.000
A-A-G	0.0030 (1)	0.0186 (2)	8.984 (0.801–100.7)	0.0904	0.211
A-A-A	0.0000 (0)	0.0064 (1)	13.39 (0.539–332.9)	0.1845	0.323

MMD, moyamoya disease; N/A, not application; FDR, false discovery rate.

Table S3. Haplotype analysis (2 site) of *RNF213* in MMD patients and control subjects of pediatric group.

Haplotype	Controls (2*n* = 204)	Age < 18 years (2*n* = 114)	OR (95% CI)	*p*	FDR-*P*
RNF213 4448G>A/4810G>A					
G-G	0.8728 (178)	0.5000 (57)	1.000 (reference)		
G-A	0.0634 (13)	0.4474 (51)	12.25 (6.217–24.14)	<0.0001	0.0002
A-G	0.0634 (13)	0.0526 (6)	1.441 (0.524–3.967)	0.580	0.580
A-A	0.0003 (0)	0.0000 (0)	N/A		
RNF213 4448G>A/4863G>A					
G-G	0.8195 (167)	0.8947 (102)	1.000 (reference)		
G-A	0.1168 (24)	0.0526 (6)	0.409 (0.162–1.035)	0.070	0.140
A-G	0.0629 (13)	0.0526 (6)	0.756 (0.278–2.051)	0.634	0.634
A-A	0.0008 (0)	0.0000 (0)	N/A		
RNF213 4448G>A/4950G>A					
G-G	0.8891 (181)	0.8924 (102)	1.000 (reference)		
G-A	0.0471 (10)	0.0549 (6)	1.065 (0.376–3.015)	1.000	1.000
A-G	0.0569 (12)	0.0374 (4)	0.592 (0.186–1.882)	0.433	0.649
A-A	0.0068 (1)	0.0152 (2)	3.549 (0.318–39.64)	0.300	0.649
RNF213 4810G>A/4863G>A					
G-G	0.8379 (171)	0.5526 (63)	1.000 (reference)		
G-A	0.0984 (20)	0.0000 (0)	0.066 (0.004–1.106)	0.005	0.008
A-G	0.0444 (9)	0.3947 (45)	13.57 (6.271–29.37)	<0.0001	0.0003
A-A	0.0193 (4)	0.0526 (6)	4.071 (1.112–14.91)	0.033	0.033
RNF213 4810G>A/4950G>A					
G-G	0.8891 (181)	0.4825 (55)	1.000 (reference)		
G-A	0.0471 (10)	0.0702 (8)	2.633 (0.990–6.999)	0.084	0.126
A-G	0.0569 (12)	0.4474 (51)	13.99 (6.962–28.10)	<0.0001	0.0003
A-A	0.0068 (1)	0.0000 (0)	1.090 (0.044–27.16)	1.000	1.000
RNF213 4863G>A/4950G>A					
G-G	0.8284 (169)	0.8772 (100)	1.000 (reference)		
G-A	0.0539 (11)	0.0702 (8)	1.229 (0.478–3.159)	0.807	0.807
A-G	0.1176 (24)	0.0526 (6)	0.423 (0.167–1.069)	0.071	0.142
A-A	0	0	N/A		

MMD, moyamoya disease; N/A, not application; FDR, false discovery rate.

Table S4. Haplotype analysis (2 site) of *RNF213* in MMD patients and control subjects of adult group.

Haplotypes	Controls (2*n* = 302)	Age ≥ 18 Years (2*n* = 120)	OR (95% CI)	*p*	FDR-*P*
RNF213 4448G>A/4810G>A					
G-G	0.9092 (275)	0.5583 (67)	1.000 (reference)		
G-A	0.0180 (5)	0.4250 (51)	41.87 (16.08–109.0)	<0.0001	0.000
A-G	0.0709 (21)	0.0000 (0)	0.095 (0.006–1.588)	0.019	0.028
A-A	0.0019 (1)	0.0167 (2)	8.209 (0.733–91.94)	0.103	0.103
RNF213 4448G>A/4863G>A					
G-G	0.9073 (274)	0.9583 (115)	1.000 (reference)		
G-A	0.0199 (6)	0.0250 (3)	1.191 (0.293–4.847)	0.728	0.728
A-G	0.0728 (22)	0.0167 (2)	0.217 (0.050–0.937)	0.033	0.066
RNF213 4448G>A/4950G>A					
G-G	0.8675 (262)	0.8333 (100)	1.000 (reference)		
G-A	0.0596 (18)	0.1500 (18)	2.620 (1.310–5.239)	0.007	0.014
A-G	0.0728 (22)	0.0167 (2)	0.238 (0.055–1.032)	0.053	0.053
RNF213 4810G>A/4863G>A					
G-G	0.9633 (291)	0.5583 (67)	1.000 (reference)		
G-A	0.0169 (5)	0.0000 (0)	0.393 (0.021–7.191)	0.589	0.589
A-G	0.0169 (5)	0.4167 (50)	43.43 (16.68–113.1)	<0.0001	0.000
A-A	0.0030 (1)	0.0250 (3)	13.03 (1.334–127.3)	0.024	0.036
RNF213 4810G>A/4950G>A					
G-G	0.9205 (278)	0.5066 (61)	1.000 (reference)		
G-A	0.0596 (18)	0.0517 (6)	1.519 (0.579–3.987)	0.414	0.414
A-G	0.0199 (6)	0.3434 (41)	31.14 (12.65–76.65)	<0.0001	0.000
A-A	0.0000 (0)	0.0983 (12)	113.2 (6.609–1939)	<0.0001	0.000
RNF213 4863G>A/4950G>A					
G-G	0.9227 (279)	0.8298 (100)	1.000 (reference)		
G-A	0.0574 (17)	0.1452 (17)	2.790 (1.371–5.676)	0.005	0.016
A-G	0.0177 (5)	0.0202 (2)	1.116 (0.213–5.846)	1.000	1.000
A-A	0.0022 (1)	0.0048 (1)	2.79 (0.1728–45.06)	0.460	0.691

MMD, moyamoya disease; N/A, not application; FDR, false discovery rate.

Table S5. PCR conditions, primers and restriction enzyme used in this study.

Gene	RefSNP(rs) Number	PCR Condition		Primer Sequence (5′-3′)	R/E
RNF213 4448	rs148731719	95 °C 15 min,<br>95 °C 20 s, −63 °C 40 s, −72 °C 30 s,<br>72 °C 5 min.	35 cycles	CAAATGGTGGTGATTTCTCCA<br>CAACAATGGCACAGAATTGTC	*Alu*I
RNF213 4810	rs112735431	95 °C 15 min,<br>95 °C 20 s, −65 °C 40 s, −72 °C 30 s,<br>72 °C 5 min.	35 cycles	AGCAGAGCTGAGGCTGGTAA<br>CTGTCAGAGCAGAGCCACAC	*Hpy*188I
RNF213 4863	rs760732823	94 °C 7 min,<br>94 °C 30 s, −64 °C 30 s, −72 °C 30 s,<br>72 °C 7 min.	35 cycles	TGTGTGTGGAGCTGATGGCT<br>AGGGAGGAGATACAGACCAGACT	*Hpy*188I
RNF213 4950	rs37144113	94 °C 7 min,<br>94 °C 30 s, −64 °C 30 s, −72 °C 30 s,<br>72 °C 7 min.	35 cycles	GGTGGAGGAGGGCAGAGAGACCGTGCACGA<br>CTTCCCTCTCTCGAGAAACACACCAA	*BssS*αI

PCR, polymerase chain reaction; R/E, restriction enzyme.
